# MSARC: Multiple sequence alignment by residue clustering

**DOI:** 10.1186/1748-7188-9-12

**Published:** 2014-04-16

**Authors:** Michał Modzelewski, Norbert Dojer

**Affiliations:** 1 Institute of Informatics, University of Warsaw

**Keywords:** Multiple sequence alignment, Stochastic alignment, Graph partitioning

## Abstract

**Background:**

Progressive methods offer efficient and reasonably good solutions to the multiple sequence alignment problem. However, resulting alignments are biased by guide-trees, especially for relatively distant sequences.

**Results:**

We propose MSARC, a new graph-clustering based algorithm that aligns sequence sets without guide-trees. Experiments on the BAliBASE dataset show that MSARC achieves alignment quality similar to the best progressive methods.

Furthermore, MSARC outperforms them on sequence sets whose evolutionary distances are difficult to represent by a phylogenetic tree. These datasets are most exposed to the guide-tree bias of alignments.

**Availability:**

MSARC is available at http://bioputer.mimuw.edu.pl/msarc

## Background

Determining the alignment of a group of biological sequences is among the most common problems in computational biology. The dynamic programming method of pairwise sequence alignment can be readily extended to multiple sequences but requires the computation of an *n*-dimensional matrix to align *n* sequences. Since the size of such a matrix is exponential with respect to *n*, the time and space complexity of this method is exponential too.

*Progressive alignment*[1] offers a substantial complexity reduction at the cost of possible loss of the optimal solution. Within this approach, subset alignments are sequentially pairwise aligned to build the final multiple alignment. The order of pairwise alignments is determined by a guide-tree representing the phylogenetic relationships between sequences.

There are two drawbacks of the progressive alignment approach. First, the accuracy of the guide-tree affects the quality of the final alignment. This problem is particularly important in the field of phylogeny reconstruction, because multiple alignment acts as a preprocessing step in most prominent methods of inferring a phylogenetic tree of sequences. It has been shown that, within this approach, the inferred phylogeny is biased towards the initial guide-tree [2,3].

Second, only sequences belonging to currently aligned subsets contribute to their pairwise alignment. Even if a guide-tree reflects correct phylogenetic relationships, these alignments may be inconsistent with remaining sequences and the inconsistencies are propagated to further steps. To address this problem, in recent programs [4-8] progressive alignment is usually preceded by *consistency transformation* (incorporating information from all pairwise alignments into the objective function) and/or followed by *iterative refinement* of the multiple alignment of all sequences. Moreover, recently several strategies avoiding guide trees altogether were also proposed [9-11].

**Figure 1:**
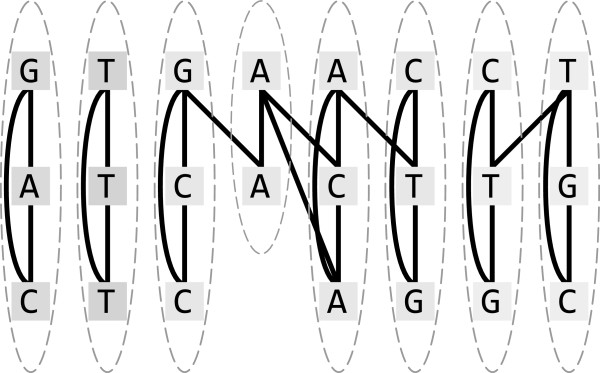
**Alignment graph and its desired clustering.** Clusters form columns of a corresponding multiple sequence alignment.

In the present paper we propose MSARC, a new non-progressive multiple sequence alignment algorithm. MSARC constructs a graph with all residues from all sequences as nodes and edges weighted with alignment affinities of its adjacent nodes. Columns of best multiple alignments tend to form clusters in this graph, so in the next step residues are clustered (see Figure 1). Finally, MSARC refines the multiple alignment corresponding to the clustering.

Experiments on the BAliBASE dataset [12] show that our approach is competitive with the best progressive methods and significantly outperforms most non-progressive algorithms. Moreover, MSARC is the best aligner for sequence sets with very low levels of conservation. This feature makes MSARC a promising preprocessing tool for phylogeny reconstruction pipelines.

## Methods

MSARC aligns sequence sets in several steps. In a preprocessing step, following Probalign [8], *stochastic alignments* are calculated for all pairs of sequences and consistency transformation is applied to resulting posterior probabilities of residue correspondences. Transformed probabilities, called residue alignment affinities, represent weights of an *alignment graph*^a^.

**Figure 2:**
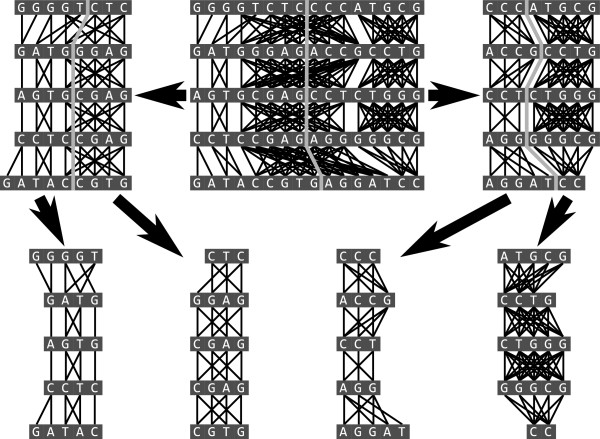
**Hierarchical divisive clustering of residues.** An alignment graph is recursively partitioned by finding a balanced minimal cut while maintaining the ordering of residues until all parts have at most one residue from each sequence. The final alignment is constructed by concatenating these parts (alignment columns) from left to right.

MSARC clusters this graph with a top-down hierarchical method (Figure 2). Division steps are based on the Fiduccia-Mattheyses graph partitioning algorithm [13], adapted to satisfy constraints imposed by the sequence order of residues. Finally, the multiple alignment corresponding to the resulting clustering is refined with the iterative improvement strategy proposed in Probcons [7], adapted to remove clustering artefacts.

### Pairwise stochastic alignment

The concept of stochastic (or probability) alignment was proposed in [14]. Given a pair of sequences, this framework defines statistical weights of their possible alignments. Based on these weights, for each pair of residues from both sequences, the posterior probability of being aligned may be computed.

A consensus of highly weighted suboptimal alignments was shown to contain pairs with significant probabilities that agree with structural alignments despite the optimal alignment deviating significantly. Mückstein et al. [15] suggest the use of the method as a starting point for improved multiple sequence alignment procedures.

The statistical weight $W\left(A\right)$ of an alignment  is the product of the individual weights of (mis-)matches and gaps [16]. It may be obtained from the standard similarity scoring function S(A) with the following formula:

(1) $$W\left(A\right)=e^{\mathrm{\beta S}\left(A\right)},$$

where *β* corresponds to the inverse of Boltzmann’s constant and should be adjusted to the match/mismatch scoring function *s*(*x*,*y*) (in fact, *β* simply rescales the scoring function).

The probability distribution over all alignments $A^{*}$ is achieved by normalizing this value. The normalization factor *Z* is called the *partition function* of the alignment problem [14], and is defined as

(2) $$Z=\underset{A\in A^{*}}{\sum }W\left(A\right)=\underset{A\in A^{*}}{\sum }e^{\mathrm{\beta S}\left(A\right)}.$$

The probability $P\left(A\right)$ of an alignment can be calculated by

(3) $$P\left(A\right)=\frac{W\left(A\right)}{Z}=\frac{e^{\mathrm{\beta S}\left(A\right)}}{Z}.$$

Let **P**(*a*_*i*_∼*b*_*j*_) denote the posterior probability that residues *a*_*i*_ and *b*_*j*_ are aligned.

We can calculate it as the sum of probabilities of all alignments with *a*_*i*_ and *b*_*j*_ in a common column (denoted by $A_{a_{i}∼b_{j}}^{*}$):

(4) $$\;\begin{array}{l} P\left(a_{i}∼b_{j}\right)\\ \;=\underset{A\in A_{a_{i}∼b_{j}}^{*}}{\sum }P(A)=\frac{\underset{A\in A_{a_{i}∼b_{j}}^{*}}{\sum }e^{\mathrm{\beta S}\left(A\right)}}{Z}\\ \;=\frac{\left(\underset{A_{i-1,j-1}}{\sum }e^{\mathrm{\beta S}(A_{i-1,j-1})}\right)e^{\mathrm{\beta s}(a_{i},b_{j})}\left(\underset{{\hat{A}}_{i+1,j+1}}{\sum }e^{\mathrm{\beta S}({\hat{A}}_{i+1,j+1})}\right)}{Z}\\ \;=\frac{Z_{i-1,j-1}\;e^{\mathrm{\beta s}\left(a_{i},b_{j}\right)}\;{\hat{Z}}_{i+1,j+1}}{Z}. \end{array}$$

Here we use the notation $A_{i,j}$ for an alignment of the sequence prefixes *a*_1_⋯*a*_*i*_ and *b*_1_⋯*b*_*j*_, and ${\hat{A}}_{i,j}$ for an alignment of the sequence suffixes *a*_*i*_⋯*a*_*m*_ and *b*_*j*_⋯*b*_*n*_. Analogously, *Z*_*i*,*j*_ is the partition function over the prefix alignments and ${\hat{Z}}_{i,j}$ is the (reverse) partition function over the suffix alignments.

An efficient algorithm for calculating the partition function can be derived from the Gotoh maximum score algorithm [17] by replacing the maximum operations with additions [14-16]:

(5) $$\begin{array}{ll} Z_{i,j}^{M} & =\left(Z_{i\;-\;1,j\;-\;1}^{M}\;+\;Z_{i\;-\;1,j\;-\;1}^{E}\;+\;Z_{i\;-\;1,j\;-\;1}^{F}\right)\;e^{\mathrm{\beta s}\left(a_{i},b_{j}\right)}\;,\; \end{array}$$

(6) $$\begin{array}{ll} Z_{i,j}^{E} & =\left(Z_{i,j-1}^{M}+Z_{i,j-1}^{F}\right)e^{\beta g_{o}}+Z_{i,j-1}^{E}e^{\beta g_{\text{ext}}}\;,\; \end{array}$$

(7) $$\begin{array}{ll} Z_{i,j}^{F} & =\left(Z_{i-1,j}^{M}+Z_{i-1,j}^{E}\right)e^{\beta g_{o}}+Z_{i-1,j}^{F}e^{\beta g_{\text{ext}}}\;,\; \end{array}$$

(8) $$\begin{array}{ll} Z_{i,j} & =Z_{i,j}^{M}+Z_{i,j}^{E}+Z_{i,j}^{F}.\; \end{array}$$

The reverse partition function can be calculated using the same recursion in reverse, starting from the ends of the aligned sequences.

We also considered a slight modification of formulas 6 and 7:

(9) $$\begin{array}{ll} Z_{i,j}^{E} & =Z_{i,j-1}^{M}e^{\beta g_{o}}+Z_{i,j-1}^{E}e^{\beta g_{\text{ext}}},\; \end{array}$$

(10) $$\begin{array}{ll} Z_{i,j}^{F} & =Z_{i-1,j}^{M}e^{\beta g_{o}}+Z_{i-1,j}^{F}e^{\beta g_{\text{ext}}}.\; \end{array}$$

In this case insertions and deletions must be separated by at least one match/mismatch position. This variant was proposed by Miyazawa [14] and applied in the Probalign [8] and MSAProbs [18] aligners.

### Alignment graphs

Let us regard probabilities **P**(*a*_*i*_∼*b*_*j*_) as a representation of a bipartite graph with weighted edges, i.e. a graph with residues from both sequences as nodes and edges joining each *a*_*i*_ with each *b*_*j*_.

Given a set *S* of *k* sequences to be aligned, we would like to analogously represent their residue alignment affinity by a *k*-partite weighted graph. It may be obtained by joining pairwise alignment graphs for all pairs of *S*-sequences. However, separate computation of edge weights for each pair of sequences does not exploit information included in the remaining alignments. Thus we decided to address this problem with a so called *consistency transformation*[4,7], successfully used in progressive methods.

In order to incorporate correspondence with residues from other sequences, MSARC re-estimates the residue alignment affinity according to the following formula:

(11) $$\;P^{\prime }\left(a_{i}∼b_{j}\right)\leftarrow \underset{c\in S}{\sum }\frac{w_{\text{ac}}w_{\text{cb}}}{\underset{c^{\prime }\in S}{\sum }w_{ac^{\prime }}w_{c^{\prime }b}}\overset{\left|c\right|}{\underset{l=0}{\sum }}P\left(a_{i}∼c_{l}\right)P\left(c_{l}\;∼\;b_{j}\right),$$

where *w*_*x* *y*_ are weights specifying the relative contribution to the transformation of a sequence pair *xy*.

If *P*_*a* *b*_ is a matrix of current residue alignment affinities for sequences *a* and *b*, the matrix form equivalent transformation is given by

(12) $$P_{\text{ab}}^{\prime }\leftarrow \underset{c\in S}{\sum }\frac{w_{\text{ac}}w_{\text{cb}}}{\underset{c^{\prime }\in S}{\sum }w_{ac^{\prime }}w_{c^{\prime }b}}P_{\text{ac}}\cdot P_{\text{cb}},$$

where · stands for matrix multiplication.

MSARC allows for two options of weight assignments. In the first one all the weights are set to 1 and the above formula simplifies to the following:

(13) $$P_{\text{ab}}^{\prime }\leftarrow \underset{c\in S}{\sum }\frac{1}{\left|S\right|}P_{\text{ac}}\cdot P_{\text{cb}}.$$

It results in the variant of consistency transformation used in Probalign [8] and ProbCons [7].

In the second option weights are calculated according to the following formula:

(14) $$w_{\text{ab}}\leftarrow \frac{\overset{\left|a\right|}{\underset{i=1}{\sum }}\overset{\left|b\right|}{\underset{j=1}{\sum }}P\left(a_{i}∼b_{j}\right)}{\min(|a|,|b|)}.$$

The idea behind the above formula is that the sum of a row/column of a matrix *P*_*a* *b*_ yields the probability that the corresponding residue is aligned to one in the other sequence (not a gap). If sequences *a* and *b* are similar, alignments with fewer gaps are preferred, so (at least for the shorter sequence) most of the sums are close to 1. Consequently, the *w*_*a* *b*_ is close to 1 as well. On the other hand, weights are much closer to 0 for pairs of dissimilar sequences.

Thus *w*_*a* *b*_ measures the similarity of sequences *a* and *b*. Therefore sequences *c* that are similar to *a* and *b* contribute to $P_{\text{ab}}^{\prime }$ more significantly than others.

The consistency transformation may be iterated any number of times, but excessive iterations blur the structure of residue affinity. Following Probalign [8] and ProbCons [7], MSARC performs two iterations by default.

### Residue clustering

**Figure 3:**
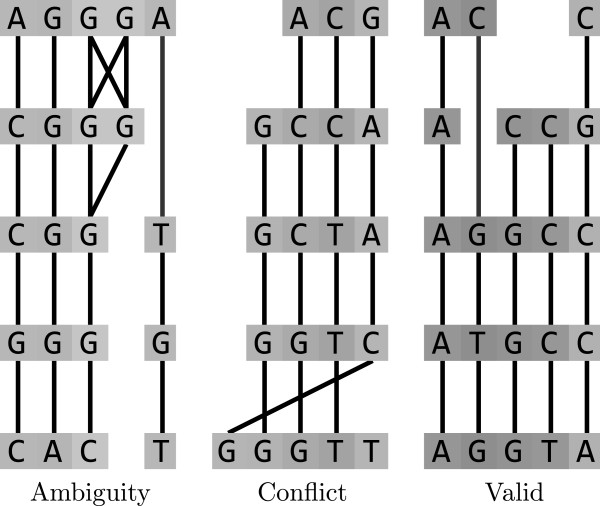
Clusterings inconsistent (left and middle) and consistent (right) with the alignment structure.

Columns of any multiple alignment form a partition of the set of sequence residues. The main idea of MSARC is to reconstruct the alignment by clustering an alignment graph into columns. The clustering method must satisfy constraints imposed by alignment structure. First, each cluster may contain at most one residue from a single sequence. Second, the set of all clusters must be orderable consistently with sequence orders of their residues. Violation of the first constraint will be called *ambiguity*, while violation of the second one – *conflict* (see Figure 3).

Towards this objective, MSARC applies top-down hierarchical clustering (see Figure 2). Within this approach, the alignment graph is recursively split into two parts until no ambiguous cluster is left. Each partition step results from a single cut through all sequences, so clusterings are conflict-free at each step of the procedure. Consequently, the final clustering represents a proper multiple alignment.

Optimal clustering is expected to maximize residue alignment affinity within clusters and minimize it between them. Therefore, the partition selection in recursive steps of the clustering procedure should minimize the sum of weights of edges cut by the partition. This is in fact the objective of the well-known problem of *graph partitioning*, i.e. dividing graph nodes into roughly equal parts such that the sum of weights of edges connecting nodes in different parts is minimized.

The Fiduccia-Mattheyses algorithm [13] is an efficient heuristic for the graph partitioning problem. After selecting an initial, possibly random partition, it calculates for each node the change in cost caused by moving it between parts, called *gain*. Subsequently, single nodes are greedily moved between partitions based on the maximum gain and gains of remaining nodes are updated. The process is repeated in *passes*, where each node can be moved only once per pass. The best partition found in a pass is chosen as the initial partition for the next pass. The algorithm terminates when a pass fails to improve the partition. Grouping single moves into passes helps the algorithm to escape local optima, since intermediate partitions in a pass may have negative gains. An additional balance condition is enforced, disallowing movement from a partition that contains less than a minimum desired number of nodes.

Fiduccia-Mattheyses algorithm needs to be modified in order to deal with alignment graphs. Mainly, residues are not moved independently; since the graph topology has to be maintained, moving a residue involves moving all the residues positioned between it and a current cut point on its sequence. This modification implies further changes in the design of data structures for gain processing. Next, the sizes of parts in considered partitions cannot differ by more than the maximum cluster size in a final clustering, i.e., the number of aligned sequences. This choice implies minimal search space containing partitions consistent with all possible multiple alignments. In the initial partition sequences are cut in their midpoints.

**Figure 4:**
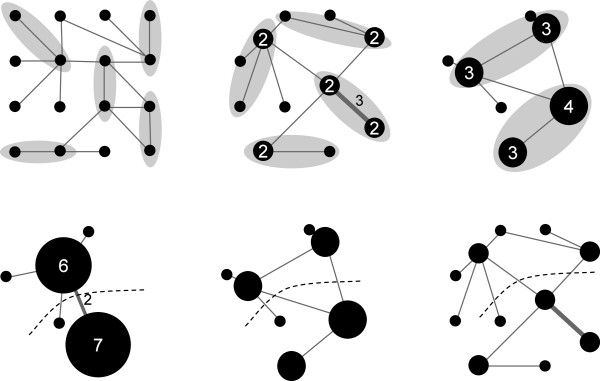
**An example of the coarsening of a graph, the partitioning of the coarse graph, and the subsequent uncoarsening of the partitioned coarse graph (without a refinement step after each iteration of uncoarsening).** Pairs of nodes selected for merging in each step of coarsening are highlighted. Initial node and edge weights are all one. Node size and edge width, and the nearby number values indicate the weights after merging.

**Figure 5:**
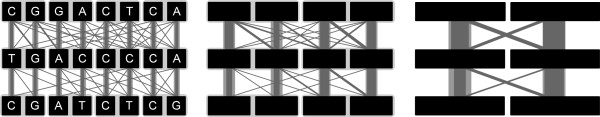
**The coarsening of an alignment graph.** Lighter colored edges represent edges between the top and bottom sequences, darker edges represent edges between neighboring sequences.

The Fiduccia-Mattheyses heuristic may be optionally extended with a *multilevel* scheme [19]. In this approach increasingly coarse approximations of the graph are created by an iterative process called *coarsening*. At each iteration step selected pairs of nodes are merged into single nodes. Adjacent edges are merged accordingly and weighted with sums of original weights. The final coarsest graph is partitioned using the Fiduccia-Mattheyses algorithm. Then the partition is projected back to the original graph through the series of *uncoarsening* operations (see Figure 4), each of which is followed by a Fiduccia-Mattheyses based refinement. Because the last refinement is applied to the original graph, the multilevel scheme in fact reduces the problem of selecting an initial partition to the problem of selecting pairs of nodes to be merged. In alignment graphs only neighboring nodes can be merged, so MSARC just merges consecutive pairs of neighboring nodes (see Figure 5).

### Refinement

**Figure 6:**
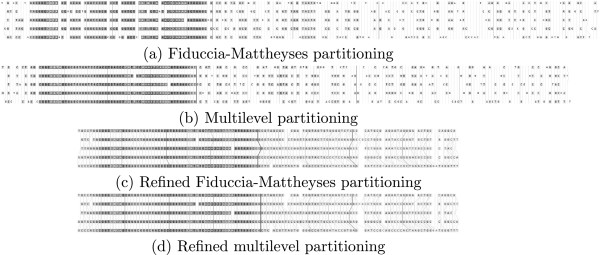
**Example visualization of the alignment produced by the graph partitioning methods alone (ab) and graph partitioning followed by refinement (cd).** Residue colors reflect how well the column is aligned based on residue match probabilities (darker is better). Partition cuts are colored to show the order of partitioning with darker cuts being performed earlier.

An example of alignment columns produced by residue clustering can be seen in Figure 6(ab). Presented alignments contain surprisingly many spaces, especially in their right parts. Some of them are clearly superfluous, e.g. in both alignments there are 3 consecutive columns near the right end, each consisting of 4 spaces and 1 G-nucleotide occupying a different row.

Therefore we decided to add a refinement step, following the method used in ProbCons [7]. Sequences are split into two groups and the groups are pairwise re-aligned. Re-alignment is performed using the Needleman-Wunsch algorithm with the score for each pair of positions defined as the sum of posterior probabilities for all non-gap pairs and zero gap-penalty. First each sequence is re-aligned with the remaining sequences, since such division is very efficient in removing superfluous spaces. Next, several randomly selected sequence subsets are re-aligned against the rest.

Figures 6(cd) show the results of refining the alignments from Figures 6(ab). Refinement removed superfluous spaces from the clustering process and optimized the alignment. Note that the final post-refinement alignments turned out to be the same for both Fiduccia-Mattheyses and multilevel method of graph partitioning.

Löytynoja and Goldman argue in [3] that progressive methods tend to force alignments of non-homologous sequence fragments inserted in corresponding locations of aligned sequences. This tendency leads to systematic errors of the downstream analyses in phylogenetic pipelines, including overestimation of substitution and deletion events. Unfortunately, iterative refinement may be one of possible source of such effects. Therefore the number of iterations in subset re-alignment step in MSARC is adjustable, in particular the whole step may be turned off.

### Computational complexity

Let *n* denote a number of sequences to align and let *l* be their maximum length. Both time and space complexities of stochastic alignment are $O(n^{2}l^{2})$.

Computations in the other steps use data structures for sparse matrices, so the complexity depends on the number *c* of non-zero values per row/column. This number depends on the cutoff parameter *t*_*c*_ (entries <*t*_*c*_ are set to 0), namely *c*≤1/*t*_*c*_. However, we observe that *c* tends to be much lower than this bound, e.g. *c* rarely exceeds 5 for the default *t*_*c*_=0.01.

MSARC implementation of consistency transformation requires $O(n^{2}c^{2}l)$ time. Space complexity of this and the remaining steps is dominated by sparse matrices and equals $O(n^{2}\text{cl})$.

The time complexity of one pass of the Fiduccia-Mattheyses algorithm on whole sequences is $O(n^{2}cl^{2})$. We observe that the algorithm converges after very few passes, but it is hard to prove a reasonable asymptotic bound. The complexity of the whole clustering is asymptoticly equal to the complexity of the main partition step.

The time complexity of iterative refinement belongs to the class $O(n^{2}cl^{2})$.

## Results

### Benchmark data and methodology

MSARC was tested against the BAliBASE 3.0 benchmark database [1]. It contains manually refined reference protein alignments based on 3D structural superpositions. Each alignment contains core-regions that correspond to the most reliably alignable sections of the alignment. Alignments are divided into five sets designed to evaluate performance on varying types of problems: 

• Equidistant sequences with two different levels of conservation 

very divergent sequences (<20*%* identity)

medium to divergent sequences (20−40*%* identity)

• Families aligned with a highly divergent “orphan” sequence

• Subgroups with <25*%* residue identity between groups

• Sequences with N/C-terminal extensions

• Internal insertions

BAliBASE 3.0 also provides a program comparing given alignments with a reference one. Alignments are scored according to two metrics. A sum-of-pairs score (SP) showing the ratio of residue pairs that are correctly aligned, and a total column (TC) score showing the ratio of correctly aligned columns. Both scores can be applied to full sequences or just the core-regions.

We decided to present results based on core-region scores only, since the corresponding sections of the reference alignments are most reliable. Moreover, results for full sequence scores are very similar.

### Benchmarking MSARC variants

Two steps of MSARC algorithm: stochastic alignment and iterative refinement follow the respective steps in Probalign [7]. Therefore we decided to set a bunch of related parameters to Probalign’s defaults. Namely, MSARC was run with Gonnet 160 similarity matrix [20], gap penalties of −22, −1 and 0 for gap open, extension and terminal gaps respectively, *β*=0.2, a cut-off value for posterior probabilities of 0.01 (values smaller than the cutoff are set to 0 and operations designed for sparse matrices are used in order to speed up computations), two iterations of the consistency transformation and 100 iterations of iterative refinement.

On the other hand, we decided to evaluate three parameters that seem to be crucial for steps specific for MSARC approach. First, residue clustering may be performed with basic or multilevel Fiduccia-Mattheyses algorithm. Second, weighted or unweighted consistency transformation may be applied. Third, stochastic pairwise alignment may be based on equations (5)-(8) (i.e. stochastic version of classical Gotoh algorithm) or equations (6) and (7) may be replaced with equations (9) and (10), respectively. The modified formula disallows consecutive insertions and deletions, as is done in Probalign and MSAProbs.

Various combinations of the above options were tested on the BAliBASE sequences. Results are presented in Table 1. The variant with neighboring insertions and deletions allowed, weighted consistency transformation and residue clustering with basic Fiduccia-Mattheyses algorithm has the best overall results, so it was selected for comparison with other methods. However, the differences are rather marginal.

**Table 1:** Evaluation of MSARC variants

**MSARC variant**				**SP/TC scores**						
**Alt. indels**	**Weighted**	**Multilevel**		**All**	**XX-XX RV11**	**XX-XX RV12**	**XX-XX RV20**	**XX-XX RV30**	**XX-XX RV40**	**XX-XX RV50**
yes	yes	no		$\frac{87.6}{57.1}$	$\frac{69.9}{46.3}$	$\frac{94.5}{85.7}$	$\frac{92.5}{39.2}$	$\frac{83.7}{47.2}$	$\frac{93.2}{62.3}$	$\frac{88.7}{51.6}$
yes	yes	yes		$\frac{87.6}{57.0}$	$\frac{69.7}{46.5}$	$\frac{94.5}{85.8}$	$\frac{92.5}{39.0}$	$\frac{83.6}{46.9}$	$\frac{93.2}{61.8}$	$\frac{88.7}{51.9}$
yes	no	no		$\frac{87.5}{56.6}$	$\frac{69.3}{45.5}$	$\frac{94.4}{85.6}$	$\frac{92.5}{39.6}$	$\frac{83.7}{47.6}$	$\frac{93.0}{61.2}$	$\frac{88.6}{49.6}$
yes	no	yes		$\frac{87.5}{56.6}$	$\frac{69.6}{45.6}$	$\frac{94.5}{85.8}$	$\frac{92.5}{39.3}$	$\frac{83.4}{47.0}$	$\frac{93.1}{61.4}$	$\frac{88.4}{49.6}$
no	yes	no		$\frac{87.5}{57.0}$	$\frac{69.2}{45.6}$	$\frac{94.4}{85.7}$	$\frac{92.5}{39.5}$	$\frac{83.5}{47.1}$	$\frac{93.2}{62.2}$	$\frac{89.0}{51.9}$
no	yes	yes		$\frac{87.5}{57.1}$	$\frac{69.2}{46.2}$	$\frac{94.4}{85.6}$	$\frac{92.5}{39.2}$	$\frac{83.7}{47.7}$	$\frac{93.2}{62.4}$	$\frac{88.7}{51.6}$
no	no	no		$\frac{87.5}{56.6}$	$\frac{69.4}{45.6}$	$\frac{94.5}{85.7}$	$\frac{92.5}{39.7}$	$\frac{83.5}{46.9}$	$\frac{93.0}{61.3}$	$\frac{88.5}{49.7}$
no	no	yes		$\frac{87.5}{56.7}$	$\frac{69.5}{45.7}$	$\frac{94.4}{85.7}$	$\frac{92.5}{39.1}$	$\frac{83.5}{47.0}$	$\frac{93.1}{61.7}$	$\frac{88.6}{49.7}$

All the combinations of the following options are evaluated: (dis-)allowing for neighboring insertions and deletions in pairwise alignments, (not) weighting sequence pairs in consistency transformation and (not) using multilevel scheme in residue clustering. Entries show the mean SP and TC scores for each alignment algorithm on the whole BAliBASE 3.0 dataset and each of its series. All scores are multiplied by 100. Best results in each column are shown in bold.

### Comparison to other aligners

MSARC results were compared to CLUSTAL Ω [1,21] ver. 1.1.0, DIALIGN-T [9] ver. 0.2.2, DIALIGN-TX [22] ver. 1.0.2, MAFFT [6] ver. 6.903, MUSCLE [5] ver. 3.8.31, MSAProbs [18] ver. 0.9.7, Probalign [8] ver. 1.4, ProbCons [7] ver. 1.12, T-Coffee [4] ver. 9.02, FSA [10] ver. 1.15.7 and PicXAA [11] ver. 1.03. All the programs were executed with their default parameters.

Table 2 shows the SP and TC scores obtained by the alignment algorithms on the BAliBASE 3.0 benchmark. The overall results show that MSARC and PicXAA substantially outperform other non-progressive methods – DIALIGN-T and FSA have SP scores lower by ∼ 10 and TC scores lower by ∼ 15. Furthermore, MSARC and PicXAA achieve accuracy similar to the progressive methods MSAProbs and Probalign – the ranges of SP and TC scores of all four programs are 0.2 and 3.6, respectively.

**Table 2:** Comparison of multiple sequence alignment methods

	**SP/TC scores**								**Computation**
**Aligner**	**All**	**XX-XX RV11**	**XX-XX RV12**	**XX-XX RV20**	**XX-XX RV30**	**XX-XX RV40**	**XX-XX RV50**	**XX-XX BB40037**	**Time**
**Non-progressive methods**									
MSARC	$\frac{87.6}{57.1}$	$\frac{69.9}{46.3}$	$\frac{94.5}{85.7}$	$\frac{92.5}{39.2}$	$\frac{83.7}{47.2}$	$\frac{93.2}{62.3}$	$\frac{88.7}{51.6}$	$\frac{98.7}{70.0}$	16:36:37
DIALIGN-T	$\frac{77.3}{42.8}$	$\frac{49.3}{25.3}$	$\frac{88.8}{72.5}$	$\frac{86.3}{29.2}$	$\frac{74.7}{34.9}$	$\frac{82.0}{45.2}$	$\frac{80.1}{44.2}$	$\frac{52.6}{0.0}$	1:13:21
FSA	$\frac{78.5}{42.1}$	$\frac{50.3}{26.9}$	$\frac{92.4}{81.8}$	$\frac{86.7}{18.7}$	$\frac{70.7}{27.6}$	$\frac{85.5}{46.2}$	$\frac{78.2}{39.8}$	$\frac{81.8}{30.0}$	35:15:34
PicXAA	$\frac{87.8}{59.4}$	$\frac{69.0}{46.3}$	$\frac{94.6}{86.2}$	$\frac{92.5}{41.6}$	$\frac{86.0}{59.8}$	$\frac{93.1}{62.4}$	$\frac{89.2}{53.0}$	$\frac{98.7}{70.0}$	5:54:18
**Progressive methods**									
CLUSTAL Ω	$\frac{84.0}{55.4}$	$\frac{59.0}{35.8}$	$\frac{90.6}{78.9}$	$\frac{90.2}{45.0}$	$\frac{86.2}{57.5}$	$\frac{90.2}{57.9}$	$\frac{86.2}{53.3}$	$\frac{61.2}{0.0}$	**12:15**
DIALIGN-TX	$\frac{78.8}{44.3}$	$\frac{51.5}{26.5}$	$\frac{89.2}{75.2}$	$\frac{87.9}{30.5}$	$\frac{76.2}{38.5}$	$\frac{83.6}{44.8}$	$\frac{82.3}{46.6}$	$\frac{52.8}{0.0}$	1:36:05
MAFFT	$\frac{86.7}{58.4}$	$\frac{65.3}{42.8}$	$\frac{93.6}{83.8}$	$\frac{92.5}{44.6}$	$\frac{85.9}{58.1}$	$\frac{91.5}{59.0}$	$\frac{90.1}{59.4}$	$\frac{56.4}{0.0}$	54:04
MSAProbs	$\frac{87.8}{60.7}$	$\frac{68.2}{44.1}$	$\frac{94.6}{86.5}$	$\frac{92.8}{46.4}$	$\frac{86.5}{60.7}$	$\frac{92.5}{62.2}$	$\frac{90.8}{60.8}$	$\frac{59.5}{0.0}$	6:43:51
MUSCLE	$\frac{81.9}{47.5}$	$\frac{57.2}{31.8}$	$\frac{91.5}{80.4}$	$\frac{88.9}{35.0}$	$\frac{81.4}{40.9}$	$\frac{86.5}{45.0}$	$\frac{83.5}{45.9}$	$\frac{48.4}{0.0}$	23:32
Probalign	$\frac{87.6}{58.9}$	$\frac{69.5}{45.3}$	$\frac{94.6}{86.2}$	$\frac{92.6}{43.9}$	$\frac{85.3}{56.6}$	$\frac{92.2}{60.3}$	$\frac{88.7}{54.9}$	$\frac{54.2}{0.0}$	4:31:41
ProbCons	$\frac{86.4}{55.8}$	$\frac{67.0}{41.7}$	$\frac{94.1}{85.5}$	$\frac{91.7}{40.6}$	$\frac{84.5}{54.4}$	$\frac{90.3}{53.2}$	$\frac{89.4}{57.3}$	$\frac{59.3}{0.0}$	6:56:32
T-Coffee	$\frac{85.7}{55.1}$	$\frac{65.5}{40.9}$	$\frac{93.9}{84.8}$	$\frac{91.4}{40.1}$	$\frac{83.7}{49.0}$	$\frac{89.2}{54.5}$	$\frac{89.4}{58.5}$	$\frac{50.9}{0.0}$	13:53:02

Columns 2-9 show the mean SP and TC scores for each alignment algorithm on the whole BAliBASE 3.0 dataset, each of its series and case BB40037. The last column presents total CPU computation time (hh:mm:ss). All scores are multiplied by 100. Best results in each column are shown in bold.

The differences between best programs are not significant in most benchmark series (see Table 3) and correspond to their structures – MSARC and PicXAA have the best results for test series RV11 and RV40, and the worst performance on RV30. Distances in RV30 families are particularly well represented by guide trees (low similarity between highly conserved subgroups) and progressive methods can benefit from it. On the other hand, series RV11 contains highly divergent sequences for which guide-tree is poorly informative, even if it represents the correct phylogeny, and RV40 contains sequences with N/C-terminal extensions which may affect the accuracy of the estimated phylogeny. These sequence families expose progressive methods to guide-tree bias.

**Table 3:** Significance of differences in BAliBASE 3.0 SP/TC scores

**Aligner**		**XX-XX RV11**	**XX-XX RV12**	**XX-XX RV20**	**XX-XX RV30**	**XX-XX RV40**	**XX-XX RV50**	**Total**
**Non-progressive methods**								
DIALIGN-T		$\frac{+8.6e-8}{+1.5e-6}$	$\frac{+7.7e-9}{+2.2e-8}$	$\frac{+1.3e-7}{+9.6e-5}$	$\frac{+2.7e-6}{+0.0024}$	$\frac{+2.1e-9}{+4.9e-8}$	$\frac{+0.00098}{+0.027}$	$\frac{+5.3e-36}{+3.6e-26}$
FSA		$\frac{+8.6e-8}{+1.2e-6}$	$\frac{+3.5e-6}{+0.00012}$	$\frac{+3.6e-8}{+1.2e-6}$	$\frac{+2.6e-6}{+8.5e-6}$	$\frac{+8.3e-9}{+1.2e-6}$	$\frac{+0.00081}{+0.021}$	$\frac{+3.6e-34}{+3.5e-27}$
PicXAA		$\frac{+0.048}{+(0.53)}$	$\frac{-(0.82)}{-(0.98)}$	$\frac{-(0.055)}{-0.018}$	$\frac{-2.8e-5}{-7.2e-6}$	$\frac{+(0.11)}{-(0.052)}$	$\frac{-(0.063)}{-(0.37)}$	$\frac{-0.0079}{-1.3e-6}$
**Progressive methods**								
Clustal Ω		$\frac{+2.6e-7}{+5.1e-5}$	$\frac{+2.4e-5}{+0.00019}$	$\frac{+0.0048}{-0.00054}$	$\frac{-0.020}{-0.00060}$	$\frac{+2.2e-6}{+(0.16)}$	$\frac{+0.017}{-(0.77)}$	$\frac{+1.1e-13}{+(0.30)}$
DIALIGN-TX		$\frac{+1.0e-7}{+1.3e-6}$	$\frac{+6.2e-8}{+4.0e-7}$	$\frac{+2.3e-7}{+0.00040}$	$\frac{+8.7e-6}{+0.038}$	$\frac{+2.8e-9}{+1.3e-7}$	$\frac{+0.0017}{+(0.066)}$	$\frac{+3.1e-34}{+9.5e-23}$
MAFFT		$\frac{+0.0031}{+(0.11)}$	$\frac{+0.00085}{+0.005}$	$\frac{-(0.64)}{-(0.052)}$	$\frac{-0.0009}{-0.0007}$	$\frac{+0.0005}{+(0.07)}$	$\frac{-(0.072)}{-(0.062)}$	$\frac{+0.028}{-(0.55)}$
MSAProbs		$\frac{+0.028}{+(0.23)}$	$\frac{-(0.90)}{-(0.67)}$	$\frac{-0.011}{-0.00032}$	$\frac{-0.00017}{-1.4e-5}$	$\frac{+(0.61)}{+0.048}$	$\frac{-0.010}{-0.0086}$	$\frac{-0.020}{-5.9e-8}$
MUSCLE		$\frac{+7.3e-6}{+0.00017}$	$\frac{+2.8e-6}{+0.00015}$	$\frac{+0.00015}{+(0.15)}$	$\frac{+(0.19)}{+(0.52)}$	$\frac{+7.6e-9}{+2.8e-6}$	$\frac{+0.010}{+(0.072)}$	$\frac{+2.9e-22}{+3.3e-12}$
Probalign		$\frac{+(0.67)}{+(0.52)}$	$\frac{-(0.63)}{-(0.88)}$	$\frac{-0.032}{-6.8e-5}$	$\frac{-0.0099}{-0.00056}$	$\frac{+(0.62)}{+(0.060)}$	$\frac{-(0.18)}{-(0.32)}$	$\frac{-0.019}{-6.0e-6}$
ProbCons		$\frac{+0.021}{+0.037}$	$\frac{+0.0042}{+(0.19)}$	$\frac{+0.028}{-(0.19)}$	$\frac{-(0.15)}{-0.010}$	$\frac{+0.00026}{+0.022}$	$\frac{-(0.12)}{-(0.17)}$	$\frac{+0.00087}{+(0.93)}$
T-Coffee		$\frac{+0.0024}{+0.016}$	$\frac{+0.0017}{+0.013}$	$\frac{+0.0075}{-(0.51)}$	$\frac{-(0.29)}{-(0.099)}$	$\frac{+9.7e-5}{+(0.29)}$	$\frac{-0.048}{-0.026}$	$\frac{+1.3e-5}{+(0.70)}$

Entries show *p*-values indicating the significance of the mean difference of SP/TC scores between MSARC and other aligners as measured using the Wilcoxon matched-pair signed-rank test. A + means that MSARC had a higher mean score while a − means MSARC had a lower mean score. Nonsignificant *p*-values (>0.05) are shown in parentheses.

**Figure 7:**
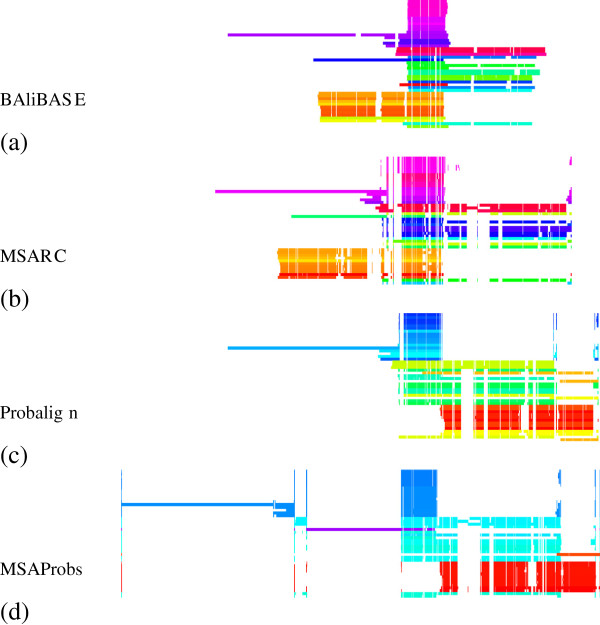
**Visualization of reference (a) and reconstructed (bcd) alignments for test case**BB40037**.** In all alignments sequences are ordered accordingly. Each sequence is colored based on the evolutionary distance to its neighbors in a phylogenetic tree, such that families of related sequences have similar colors. Trees for **(a)** and **(b)** are computed with the PhyML 3.0 program [23], using the maximum parsimony method. Trees for **(c)** and **(d)** are the guide-trees used by those aligners.

**Figure 8:**
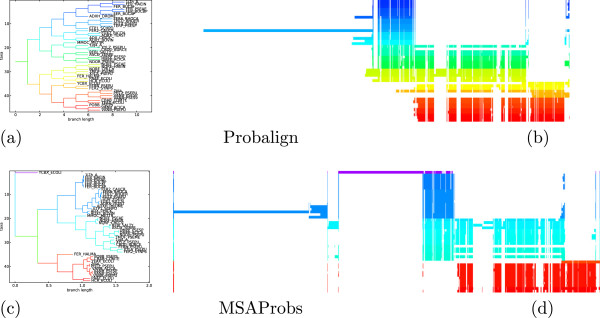
**Guide trees (ac) and alignment visualizations (bd) for test case **BB40037**and programs Probalign (ab) and MSAProbs (cd).** Tree branches and aligned sequences are colored based on the evolutionary distances to their neighbors, as computed from the guide-trees used during alignment. Sequences in alignments are ordered following their order in trees, so related sequences have similar color and are positioned together.

We illustrate this observation with an example of test case BB40037. As is shown in column 9 of Table 2, MSARC outperforms progressive methods by a large margin. The TC scores of zero means that each alignment method has shifted at least one sequence from its correct position relative to the other sequences. Figure 7 presents the structure of the reference alignment, as well as alignments generated by MSARC, Probalign and MSAProbs. The large family of red, orange and yellow colored sequences near the bottom has been misaligned by the progressive methods. The reason for this is more visible in Figure 8, where sequences in alignments are reordered according to related guide-trees.

Probalign aligns separately the first half of the sequences (blue and green) and the second half of the sequences (from yellow to red). Next, the prefixes of the second group are aligned with the suffixes of the first group, propagating an error within a yellow sub-alignment.

MSAprobs aligns separately the dark blue, light blue and red sequences. Next the blue sub-alignments are aligned together. The resulting alignment has erroneously inserted gaps near the right ends of dark blue sequences. This error is propagated in the next step, where the suffix of the blue alignment is aligned with the prefix of the red alignment. Finally, the single violet sequence is added to the alignment, splitting it in two.

For both programs, alignment errors introduced in the earlier steps are propagated to the final alignment. On the other hand, the non-progressive strategy used in MSARC yields a reasonable approximation of the reference alignment (see Figure 7(ab)).

## Conclusions

The progressive principle has dominated multiple alignment algorithms for nearly 20 years. Throughout this time, many groups have dedicated their effort to refine its accuracy to the current state. Other approaches were omitted due to high computational complexity and/or unsatisfactory quality. However, recently several non-progressive methods were proposed. Two of them, PicXAA and MSARC proved to be competitive with the best progressive approaches. Moreover, both programs outperform progressive methods on sequence sets with evolutionary distances that are difficult to represent by a phylogenetic tree.

Despite the algorithmic novelty, the non-progressive approaches to multiple alignment are interesting preprocessing tools for phylogeny reconstruction pipelines. The objective of these procedures is to infer the structure of a phylogenetic tree from a given sequence set. Multiple alignment is usually the first pipeline step. When alignment is guided by a tree, the reconstructed phylogeny is biased towards this tree. In order to minimize this effect, some phylogenetic pipelines alternately optimize a tree and an alignment [24-26]. The unbiased alignment process of MSARC may simplify this procedure and improve the reconstruction accuracy, especially in the most problematic cases.

MSARC has also the potential for quality improvements. Alternative methods of computing residue alignment affinities could be used to improve the accuracy of both MSARC and Probalign based methods. Other approaches to alignment graph partitioning may also lead to improvements in the accuracy of MSARC, for example a better method of pairing residues for multilevel coarsening than the currently used naive consecutive neighbors merging.

The main disadvantage of MSARC is its computational complexity, especially in the case of the multilevel scheme variant (MSARC is ∼2.5× slower than MSAProbs, the MSARC variant with multilevel scheme is even slower). This is the cost of avoiding the progressive approach.

## Endnotes

^a^ Our notion of alignment graph slightly differs from the one of Kececioglu [27]: removing edges between clusters transforms the former into the latter.

## Competing interests

The authors declare that they have no competing interests.

## Authors’ contributions

ND designed the overall algorithm, participated in its evaluation and wrote the manuscript. MM designed and adapted algorithmic solutions, implemented the method and participated in its evaluation. Both authors read and approved the final manuscript.
